# Germ Cell Derivation from Pluripotent Stem Cells for Understanding In Vitro Gametogenesis

**DOI:** 10.3390/cells10081889

**Published:** 2021-07-26

**Authors:** Tae-Kyung Hong, Jae-Hoon Song, So-Been Lee, Jeong-Tae Do

**Affiliations:** Department of Stem Cell and Regenerative Biotechnology, Konkuk Institute of Technology, Konkuk University, 120 Neungdong-ro, Gwangjin-gu, Seoul 05029, Korea; htk0518@gmail.com (T.-K.H.); gnswp1911@gmail.com (J.-H.S.); dlthqlsaw@gmail.com (S.-B.L.)

**Keywords:** germ cell, gametogenesis, pluripotent stem cell, infertility

## Abstract

Assisted reproductive technologies (ARTs) have developed considerably in recent years; however, they cannot rectify germ cell aplasia, such as non-obstructive azoospermia (NOA) and oocyte maturation failure syndrome. In vitro gametogenesis is a promising technology to overcome infertility, particularly germ cell aplasia. Early germ cells, such as primordial germ cells, can be relatively easily derived from pluripotent stem cells (PSCs); however, further progression to post-meiotic germ cells usually requires a gonadal niche and signals from gonadal somatic cells. Here, we review the recent advances in in vitro male and female germ cell derivation from PSCs and discuss how this technique is used to understand the biological mechanism of gamete development and gain insight into its application in infertility.

## 1. Introduction

Mammalian cells are divided into somatic and germ cells according to their roles. Somatic cells are involved in the homeostasis and survival of the body, whereas germ cells are dispensable for survival; however, they are needed only for reproduction. The ancestors of germ cells are unipotent and undergo a process called gametogenesis, to differentiate into either spermatozoa or oocytes. In vivo gametogenesis is a very sophisticated process that is difficult to recapitulate in an in vitro system. The development of stem cell differentiation protocols has made it possible to obtain many functional cell types in vitro, including somatic and germ cells. In particular, since approximately two decades, in vitro gametogenesis from pluripotent stem cells (PSCs) has been focused on in developmental biology and stem cell research [1,2]. In mice, PSCs can differentiate into functional germ cells, which can be fertilized and developed to term after implantation [3,4]. However, mature germ cells capable of forming embryos after fertilization have yet to be derived from human PSCs.

Studies on human germ cells generally involve ethical issues. The limited use of human origin materials for research impedes the identification of mechanisms of human germ cell development. Therefore, information regarding human germ cell development pathways has been deduced from the data obtained from animal models. The in vitro PSC differentiation approach can overcome the difficulties of using human materials and provide insight into the mechanism of human gametogenesis in vivo and in vitro [2].

According to the United Nations World Population Prospects, the global fertility rate is continuously declining. The current fertility rate in 2021 is 2.438 births per woman, which is a 0.41% decline compared to that in the 2020 data of 2.448 births per woman [5]. Although the etiology of infertility varies, most cases of infertility originate from the poor production of sperm or eggs. As the donation of gametes poses various ethical and legal issues, gamete derivation via differentiation of patient-origin PSCs is considered a feasible option for solving infertility without gamete donation [6]. If functional gamete formation from human PSCs were possible, male and female infertility, such as non-obstructive azoospermia and oocyte maturation failure syndrome, would be curable.

## 2. Germ Cell Differentiation Pathway in Mammals

To obtain better knowledge regarding in vitro gametogenesis, the in vivo process of germ cell formation and differentiation must be understood. Most studies of germline development have been conducted in mouse models, whose major events are shared with humans, although they differ in detail [6].

### 2.1. Specification and Migration of Primordial Germ Cells (PGCs)

**Figure 1:**
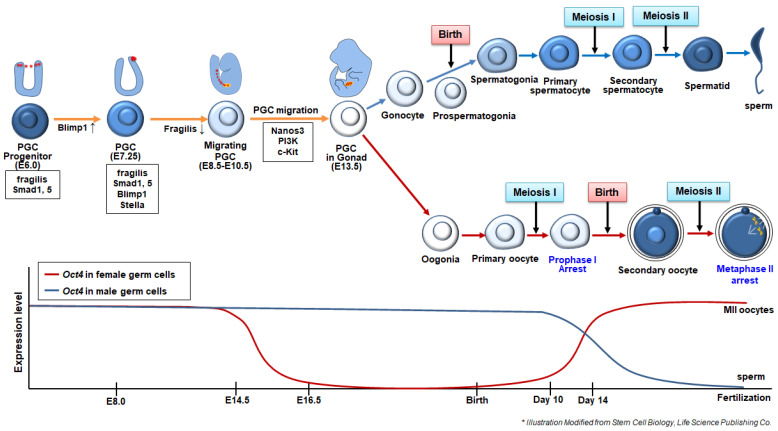
Summary of in vivo developmental process for the formation of male and female germ cells and the expression of germ cell-related genes during the germ cell development. Indicated time corresponds to the stages of mouse development. Modified from Stem Cell Biology, Life Science Publishing Co. [27].

In mice, germ cells emerge from the proximal epiblast at around E6.0, in which the precursors of primordial germ cells (PGCs) are primed to become PGCs [7] (Figure 1). Specification of PGCs is induced by signals from the extraembryonic ectoderm, such as bone morphogenetic protein 4 (BMP4), 2 (BMP2), and 8 (BMP8). These proteins bind to the receptor of proximal epiblast cells, which induces a small population of proximal epiblast cells to express *Blimp1*, also known as *PR domain zinc finger protein 1* (*Prdm1*) [6]. Blimp1 acts as a somatic program repressor. BMP-reactive epiblast cells are committed to the germ cell lineage; therefore, Blimp1 is a critical PGC determinant [8]. PGC precursors move to the posterior region of the proximal epiblast from E6.5, reaching the extraembryonic mesoderm of the posterior amniotic fold, where a cluster of founder PGCs (approximately 40–45 cells) is formed at E7.25. The founder PGCs express *Blimp1*, *Fragilis*, and *Stella* [7]. At E8.5, PGCs downregulate *fragilis* and initiate their migration to genital ridges through the hindgut. During hindgut migration, PGCs express *nanos3*, which may support the proliferation and suppress apoptosis of migrating PGCs [9]. PGCs reach genital ridges from E10.5–E13.5, in mice [10]. During this stage, PGC proliferation and migration are known to be regulated by phosphatidylinositol 3 kinase (PI3K), which is regulated by c-Kit, a cell surface receptor, and its ligand, Kit (also known as a stem cell factor or steel factor) [11]. During migration, PGCs undergo dramatic epigenetic reprogramming, such as DNA demethylation, erasure of genomic imprinting, and histone modification [12]. Resetting the epigenetic state is crucial for subsequent germ cell differentiation, as new epigenetic marks must be rewritten in the genome of newly generated gametes depending on the sex of gametes. Moreover, epigenetic profile modifications during PGC specification show species-specific aspects, particularly in the case of variations in histone methylation and acetylation kinetics, which are thoroughly affected during reprogramming of mouse and human PGC-like cells [13].

Once PGCs reach the genital ridge, where the number of PGCs is approximately 25,000, they stop migration and associate with gonadal somatic cells, followed by sex-specific morphogenesis and development. Before birth, male PGCs develop into gonocytes and prospermatogonia, whereas female PGCs develop into primary oocytes arrested in the prophase of meiosis I (prophase I) [14,15,16,17]. Shortly after birth, prospermatogonia become spermatogonia, which develop into primary spermatocytes, secondary spermatocytes, spermatids, and mature spermatozoa at puberty. Moreover, at puberty, primary oocytes develop into secondary oocytes that are arrested at the metaphase of meiosis II (MII).

During PGC specification and migration, dynamic alterations of molecular signatures are accompanied spatiotemporally. The expression of *Oct4* and *Nanog* is crucial during embryonic and germ cell development. Oct4 and Nanog are epiblast-determining factors in preimplantation stage embryos [18]. During gastrulation, *Oct4* expression is restricted to the PGCs (Figure 1). *Oct4* expression is maintained during the migration of PGCs and in spermatogonia; however, it is silenced once spermatogenesis begins [19,20]. In females, PGCs downregulate *Oct4* at the onset of meiosis at around E14.5; therefore, primary oocytes at birth do not express *Oct4*. Silenced *Oct4* is re-expressed while primary oocytes enter the growth phase (at 14 days postpartum) and develop into MII stage oocytes (Figure 1). PGCs utilize different regulatory elements, such as distal and proximal enhancers, for the expression of *Oct4* during maturation. Early migratory PGCs (at E9.5) mainly utilize distal enhancers for *Oct4* expression; however, both enhancers are used for the control of *Oct4* expression when PGCs get close to the genital ridge [21]. *Nanog* was not expressed during the early PGC specification, although it was re-expressed at the beginning of migration at E8.0. *Nanog* expression is silenced again at the spermatogonia stage in males and at the onset of meiosis in females [22,23].

In humans, similar to that in mice, BMP4 is an initiation signal for PGC specification, and the human PGC (hPGC) population is known to appear during 2–3 weeks of pregnancy (Wk) at the yolk sac endoderm [24]. hPGCs initiate migration from 4 Wk and reach genital ridges at 5–6 Wk [25,26]. hPGCs further develop into oogonia at 9 Wk and primary oocytes at 14 Wk by interaction with gonadal somatic cells [24]. Oogonia develop into primary oocytes that grow in primordial follicles and their number reaches approximately 300,000 at birth. However, the precise in vivo spatiotemporal mechanisms of early human germ cell development are not well understood because of the difficulty in accessing early human embryos [24]. In vitro differentiation of PSCs into PGCs or oocytes may be an alternative approach to gaining insights into early germ cell development in humans.

### 2.2. Sex Determination and Maturation of Germ Cells

Sex determination, whether male or female, depends on the sex of somatic cells of the genital ridge rather than the innate sex chromosomes of germ cells themselves. In mice, indifferent gonads start dimorphic patterns of development according to the sex of embryos by E13.5 [14]. In male embryos, *Sry* encoded on the Y chromosome is expressed in gonadal somatic cells from E10.5 and E12.5 [28]. *Sox9*, upregulated by Sry, induces the gonadal somatic cells into Sertoli cells, at which time the Mullerian ducts degenerate [29]. Therefore, mice deficient in these male-determining genes, *Sry* or *Sox9*, develop as female mice even if they have a Y chromosome [28,30]. Furthermore, there are several known female-determining genes, including *Wnt4*, *Dax1*, *Rspo1*, and *Foxl2*; *Dax1* is on the X chromosome, whereas the others are in autosomes [31,32]. Overexpression of these genes could result in XY femaleness [31,32,33,34] and deletion of these genes could result in ovarian dysgenesis and infertility [34,35,36].

The mesonephric duct adjacent to the genital ridge produces retinoic acid, which acts as a critical regulatory factor responsible for inducing meiosis in germ cells [37]. RA is known to induce the expression of *Stra8*, which triggers meiosis during spermatogenesis and oogenesis [38]. While males and females share similar pathways during PGC specification and premeiotic maturation, the later stages of maturation for gametogenesis differ significantly. In males, PGCs differentiate into spermatogonia, which is stopped during mitosis and present in small numbers [37]. At the onset of puberty, spermatogonia continuously divide symmetrically for self-renewal and asymmetrically for spermatogenesis [39]. Spermatogonia differentiate into primary spermatocytes, which are still diploid and begin meiosis I. Once meiosis occurs, primary spermatocytes divide into haploid secondary spermatocytes, which differentiate into spermatids containing half the DNA content. Spermatids develop into spermatozoa through spermiogenesis; they become tail-containing shapes and their DNA becomes more compact as histones are replaced by protamines [40,41]. After birth, primary oocytes remain in the resting phase at prophase I and last for decades within primary follicles until puberty [42]. At the onset of puberty, primary follicles develop into secondary follicles by stimulation with the follicle-stimulating hormone (FSH) and luteinizing hormone (LH) secreted from the anterior pituitary. In addition to FSH and LH, steroid hormones synthesized by follicular cells also cause follicular development in a paracrine and autocrine manner. Mature oocytes arrested in MII complete meiosis II after fertilization with spermatozoa and undergo embryonic development [42,43].

## 3. Germ Cell Differentiation from Pluripotent Stem Cells In Vitro

### 3.1. In Vitro Derivation of PGC-Like Cells (PGCLCs) from Pluripotent Stem Cells

In 2009, Ohinata et al. established the basic conditions for directed PGC specification in mouse pluripotent epiblast cells [44]. Pregastrula stage epiblasts were treated with BMP4, BMP8b, SCF, EGF, and LIF to induce the expression of Blimp1 and Prdm14. These epiblast-derived PGC-like cells (epiPGCs) expressing *Blimp1*-mVenus and *Stella*-ECFP showed genetic and epigenetic characteristics consistent with in vivo PGCs. When these epiPGCs were transplanted into neonatal testes, they developed into mature spermatozoa, which could fertilize oocytes and give rise to healthy offspring [44]. This research showed the basis of signal interactions essential for PGC specification from pluripotent cells and proved that the epiblast is the direct precursor of PGCs. Ohinata’s study prompted researchers to use PSCs to derive PGC-like cells (PGCLCs) in vitro, since PSCs are the in vitro derivatives of the peri-implantation epiblast.

Initial studies on in vitro germ cell specification from PSCs have mainly focused on the derivation of PGCs through embryoid body (EB) formation from ESCs or EGCs [1,45,46]. Culture in the absence of LIF and other growth factors associated with self-renewal [1] and culture with BMP4 [47] or retinoic acid [48] could induce the differentiation of PSCs into PGC-like cells (PGCLCs). Toyooka et al. isolated PGCLCs expressing Mvh (DEAD box helicase 4) during PGC differentiation and cocultured them with BMP4-producing cells for 1 d, followed by aggregation with gonadal cells isolated from E12.5 to 15.5 mice [46]. Transplantation of these aggregates into the testis capsule resulted in the formation of testicular tubules, wherein ESC-derived mature sperm were formed. Geijsen et al. also suggested that differentiation through EB formation resulted in meiotic male germ cells that could fertilize oocytes and develop the blastocyst stage [48]. However, since these germ cell derivation protocols were random differentiation strategies using EB formation, the PGC specification was very heterogeneous and inefficient (approximately 0.5–3.6% per EB), raising the need for a directed induction method for PGC specification. To increase the PGC specification from PSCs, PSCs should be maintained in a homogenous population that is germline competent. Ying et al. suggested that germline competent naïve PSCs could be maintained under culturing with MAPK and GSK inhibitors (referred to as 2i) and LIF, called the ground state [49]. The ground state of pluripotency is equivalent to that of the E4.5 epiblast of preimplantation blastocysts in gene expression patterns. Thus, the 2iL condition in mice enables the induction of germline competent ESCs/iPSCs from all mouse strains tested [49,50,51].

Hayashi et al. first proposed the stepwise differentiation of mouse PSCs into PGCLCs in 2011 [3]. They showed that mouse PSCs in ground state pluripotency were differentiated into epiblast-like cells (EpiLCs), an intermediate precursor for PGC formation, in the presence of activin A and bFGF for 2 days [3]. After culturing under conditions similar to those in Ohinata’s experiment (derivation of epiblast to PGCLCs), EpiLCs were subsequently differentiated into PGCLCs [51]. Global transcriptional profiles revealed that specification of PGCLCs from EpiLCs recapitulated the in vivo specification of epiblast to PGCs, suggesting that EpiLCs were germline competent precursors corresponding to pre-gastrulating epiblasts at around E5.75, and PGCLCs had properties similar to those of E9.5 PGCs [3]. Transplanting these male or female PGCLCs into germ cell-deficient mice results in the formation of a gonad-like structure containing gamete-like cells. In males, PGCLCs were further differentiated in an in vivo environment, such as seminiferous tubules of W/Wv mice lacking endogenous sperm, in which PGCLCs could resume spermatogenesis [3,52]. PGCLC-derived sperm developed in host testes could give rise to fertile offspring after intracytoplasmic injection (ICSI) into wild-type oocytes and transplantation into surrogate mothers [3]. Similar to the in vitro derivation of sperm, female ovary-like structures and oocyte-like cells could be derived from EpiLC-derived PGCLCs, which were differentiated from mouse ESCs and iPSCs [4]. Female PGCLCs also develop into oocytes, contributing to fertile offspring after in vitro maturation and fertilization with normal sperm [4]. These in vitro (EpiLC and PGCLC formation) and in vivo (transplantation of PGCLCs into host gonads) conjugated methods serve as bridges for the complete in vitro gametogenesis technology and provide important information for signaling cascades during germ cell fate transition.

Human PSCs can differentiate into human PGCLCs (hPGCLCs) spontaneously [53,54] or under defined culture conditions [55,56]. Gkountela et al. differentiated hESCs via serum-induced random differentiation in EBs for 9 days and sorted TRA-1-81^+^/cKIT^+^ hPGCLCs (0.3% per sorted living population), which also expressed *BLIMP1* and *OCT4* with or without *NANOS3* expression [53]. Kee et al. used another germ cell marker, VASA-GFP reporter, for hPGCLC isolation from differentiating hESCs [54]. VASA-GFP^+^ cells expressed other early germ cell markers, *DAZL*, *BLIMP1*, and *STELLA*. They also suggested that overexpression of BOULE alone or together with DAZL could enhance hPGCLC specification and meiotic progression, suggesting the importance of BOULE and DAZL for germ cell progression in humans [54]. For hPGCLC enrichment, Yu et al. treated the EBs formed from hESCs and iPSCs with activin A, BMP4, and retinoic acid, by which PGCLCs expressing DDX4, folliculogenesis-related markers, and postnatal oocyte-specific markers were derived [57]. Aggregates of PGCLCs with human granulosa cells gave rise to ovarian follicle-like structures in xenografted mice [57].

Irie et al. suggested a defined culture condition for the specification of hPGCLCs from hESCs and hiPSCs. To efficiently derive hPGCLCs, they used distinct states of PSCs that were preconditioned in a medium containing four inhibitors (4i), including CHIR99021 (GSK-3 inhibitor), PD0325901 (MEK inhibitor), SB203580 (p38 MAPK inhibitor), and SP600125 (JNK inhibitor), sustaining cells in near ground state pluripotency and aiding in easy differentiation into germ cell lineage [55]. To induce hPGCLCs, hPSCs in 4i medium were cultured in bFGF/TGFβ-containing medium for 2 days, followed by a suspension culture in the presence of BMP2 (or BMP4), LIF, stem cell factor (SCF), and epidermal growth factor (EGF). The derived hPGCLCs were similar to human PGCs (hPGCs) in terms of gene expression and epigenetic patterns. They showed that SOX17, which is known as a transcription factor for endoderm specification, might be important for hPGCLC specification and maintenance since SOX17 began to be expressed as early as day 1 after suspension culture and was associated with BLIMP1, both of which are highly expressed in hPGCs; BLIMP alone repressed somatic differentiation and enhanced germ cell specification together with SOX17 [55]. Although the preconditioned hPSC state was sufficient for producing hPGCs, Sasaki et al. pointed out that hPSCs kept in 4i medium were not genuinely at the ground state, as cells did not show consistent upregulation in naïve pluripotency markers [56]. Instead, Sasaki et al. proposed the induction of PGCs from primed hPSCs by introducing new cell types, incipient mesoderm-like cells (iMeLCs), as intermediate cells in the process of inducing hPGCLCs from hiPSCs [56]. Double transgenic hiPSCs bearing *BLIMP1*-*tdTomato* and *TFAP2C*-*EGFP* were used as reporters for human fetal germ cells [58,59]. As a first step for germ cell differentiation, hiPSCs were stimulated with activin A and CHIR99021, which produced iMeLC-expressing genes for pluripotency and the mesoderm (*T*, *EOMES*, *SP5*, and *MIXL1*). After suspension culturing with BMP4, SCF, EGF, and LIF for 8 days, these iMeLCs were converted into hPGCLCs (*BLIMP1*-*2A*-*tdTomato*^+^ and *TFAP2C*-*2A*-*EGFP*^+^*)* expressing early human germ cell markers, such as OCT4, NANOG, BLIMP1, TFAP2C, and SOX17. GO term analysis after RNA sequencing revealed that potential regulators of hPGCLC specification from iMeLCs include *BLIMP1* and *SOX17*, which were also suggested by Irie et al. [55,56].

hPGCLC derivation using hESCs was facilitated by the stimulation of activin A [60] or by adding vitamin C to the induction medium [61]. Activin A is also a well-known inducer of fetal and postnatal oogenesis, indicating that it is involved in germ cell development [62]. Epigenetic changes induced by vitamin C through enhancing the expression of ten-eleven translocation (TET) may positively affect germ cell differentiation [61].

### 3.2. In Vitro Derivation of Spermatogonia and Oogonia from Pluripotent Stem Cells

PSC-derived PGCLCs were injected into neonatal testes to generate mature sperm from PSCs. However, PGCLCs are not suitable cell types for transplantation into the testis, since the niche for PGCs is not the postnatal testis, but the prenatal proximal epiblast, hindgut, and genital ridge. After birth, the earliest germ cell types in the testis are spermatogonia that reside in the basement membrane of the seminiferous epithelium. These spermatogonia are male germ line stem cells, so-called spermatogonial stem cells (SSCs), which continuously self-renew and differentiate into spermatocytes and haploid gametes by passing between two Sertoli cells [39]. Currently, long-term in vitro culture of SSCs is possible, although PGCs cannot be cultured in vitro [63,64]. Undifferentiated SSCs established in an in vitro culture system expressed *Vasa*, *Pax7*, *Plzf*, *Gfra1*, *Etv5*, and *Bcl6b* [65]. In contrast, female germline stem cells, or oogonia, transiently exist (between E12.5 and E13.5) and undergo meiosis during embryonic development [66]. Thus far, an in vitro culture of oogonia is not possible.

Nayernia et al. first attempted to generate in vitro-derived SSCs using *Stra8*-*EGFP* and *Prm1*-*DsRed* double transgenic mouse ESCs [67]. They suggested that Stra8-EGFP^+^ cells were ESC-derived SSCs and that Prm1-DsRed^+^ could differentiate into haploid germ cells. However, Stra8-EGFP^+^ cells express PGC-specific genes; therefore, this differentiation protocol may yield heterogeneous germ cell populations, including SSCs. In addition, the ESC-derived spermatids they suggested are not fully characterized; therefore, it is not clear whether the cells are true spermatids. Ishikura et al. generated well-characterized in vitro-derived SSCs differentiated from PSCs using reconstituted testes formed by aggregating ESC-derived PGCLCs with E12.5 testicular somatic cells [68]. Aggregates of PGCLCs and testicular somatic cells formed seminiferous tubule-like structures, in which PLZF-positive SSC-like cells were detected within 21 days of culture [68]. SSC-like cells showed similar gene expression profiles and DNA methylomes to those of in vivo SSCs. In addition, after transplantation of these SSC-like cells into adult W/W^v^ mouse testes instead of neonatal testes, they developed into spermatids and spermatozoa, which could form embryos after an injection into oocytes and developed to term after transfer of the embryos to the surrogate mother.

In human, Sasaki and colleagues et al. generated prospermatogonia-like cells from hiPSCs through long-term culturing of the xenogeneic aggregates of hiPSC-derived human germ cells with mouse testicular somatic cells [69]. hPGCLCs were obtained from xenogeneic aggregates after 14 days of culture on an air–liquid interface (ALI) system where xenogeneic aggregates were cultured on the surface of the porous filter. In the ALI culture system, hiPSC-derived hPGCLCs were differentiated into multiplying (M)-prospermatogonia on day 77 and finally differentiated into primary transitional (T1)-prospermatogonia at day 120. Although they did not test the functionality of the hiPSC-derived hPGCLCs, single-cell RNA sequencing analysis revealed that the gene expression patterns of in vivo M- and T1-prospermatogonia were very similar to those of in vivo M- and T1-prospermatogonia, respectively. As hiPSC-derived T1-prospermatogonia expressed a fraction of genes associated with “spermatogenesis” and “spermatogenic failure” these cells can be used as an in vitro platform for research on the mechanism of human spermatogenesis and male infertility [69].

Recently, human female germline stem cells (oogonia) were generated in vitro from hiPSCs (*BLIMP1*-*tdTomato* and *TFAP2C*-*EGFP* double transgenic) using Sasaki’s approach, by which hiPSC-derived hPGCLCs were generated via iMeLCs [56,70]. BLIMP1-tdTomato^+^/TFAP2C-EGFP^+^ hPGCLCs were sorted by fluorescence-activated cell sorting (FACS) and cultured for further specification in xenogeneic ovary structures formed by aggregation of hiPSC-derived hPGCLCs with mouse ovarian somatic cells, as in a prospermatogonia-like cell-inducing protocol [69]. At day 7 after culture, aggregates began to form cyst-like structures and TFAP2C-EGFP^+^ cells developed into oogonia-like cells, which expressed DAZL and DDX4 and were delineated by mouse granulosa cells at culture day 77. On culture day 120, BLIMP1-tdTomato^+^/TFAP2C-EGFP^+^ cells appeared to differentiate into BLIMP1-tdTomato^+^/TFAP2C-EGFP^-^ cells, which correspond to fetal germ cells expressing genes for meiosis initiation (*STRA8*) except for meiotic recombination (*SPO1l*, *PRDM9*, *DMC1*, or *SYCP1*). Transcriptome assays revealed that hiPSC-derived oogonia-like cells (TFAP2C-EGFP^+^) were similar to those of week 7 and 9 oogonia and gonocytes of human embryos [70,71].

These reports suggest that male and female germline stem cells can be established from PSCs in vitro within reconstituted testes and ovary structures, where embryonic gonadal somatic cells were aggregated with PSC-derived PGCLCs. Of note, in vitro oogonia may provide a useful tool for elucidating the mechanism of female germline stem cell development and female infertility, which has not been studied well due to ethical issues and limitations in using human-origin materials.

### 3.3. In Vitro Derivation of Haploid Spermatid-Like Cells from Pluripotent Stem Cells

Haploid spermatid-like cells were also derived from mouse ESCs using the aggregation method [72]. Zhou et al. isolated Blimp1-Venus^+^ and Stella-ECFP^+^ PGCLCs, aggregated with testicular somatic cells in a medium containing retinoic acid for meiotic induction and BMP-2/4/7 and activin A for germ cell development and self-renewal. Under meiotic-inductive signals, including retinoic acid, BMP2/4/7, and activin A, PGCLCs in the aggregates developed beyond PGCs and underwent meiosis, confirmed by the formation of chromosome synapsis. Furthermore, hormonal stimulation, such as the follicle-stimulating hormone (FSH), testosterone, and bovine pituitary extract, induces post-meiotic haploid spermatid-like cells (SLCs) containing distinct acrosome structures and imprinted patterns in *H19* and *Snrpn* loci. After intracytoplasmic injection of SLCs into normal oocytes, fertilized embryos were successfully developed. However, to date, mature spermatozoa have not yet been derived in vitro from PSCs. Thus, in vitro spermiogenesis from haploid spermatids is currently the only obstacle in the process of complete in vitro male gametogenesis, from PSCs to mature spermatozoa.

### 3.4. In Vitro Oogenesis from Pluripotent Stem Cells

Female germ cells were formed within ovarian follicles, which are an in vivo niche for the maturation of female gametes. One follicle contains one meiotically arrested oocyte surrounded by multilayered granulosa cells at puberty [73]. During follicular maturation in vivo, primary oocytes complete meiosis I, begin meiosis II, and arrest at the metaphase of meiosis II (MII). Primordial follicles can mature in vitro by stimulating oocyte growth and meiotic competency by EGF [74] and cAMP [75,76], respectively. In vitro oogenesis could also be recapitulated in PGCs obtained from E12.5 gonads by stimulation of follicle formation using α-MEM-based medium supplemented with fetal bovine serum (FBS) and an estrogen-receptor antagonist (ICI 187, 780) [77]. These studies prompted an alternative method to derive female gametes from PSCs, where germline specification is not started.

The first published report showing germ cell differentiation from PSCs in vitro was on female gamete derivation from mouse ESCs [1]. Hübner et al. generated oocyte-like structures, which were randomly formed through EB formation, followed by two-dimensional culture. These non-directed differentiation protocols, or random differentiation, allowed even male ES cells to differentiate into oocyte-like structures. Similarly, Lacham-Kaplan et al. obtained presumptive ovary-like structures from male ESC-derived EBs using the conditioned medium from neonatal testis cells. The obtained structures contained oocyte-like cells expressing oocyte markers (*Fig*-*α* and *ZP3*), indicating the possible production of female germ cells from male PSCs [78]. However, these results were inconsistent with the proposed methods and lacked demonstration of functionality [48]; thus, further research is required. As previously mentioned, Hayashi et al. showed that in vitro-derived PGCLCs could further differentiate into functional oocytes in reconstituted ovaries after transplantation into female mice [4]. Combining these methods, Hayashi and colleagues, in 2016, finally derived MII oocytes from mouse ESCs in an entirely in vitro system [79]. They derived EpiLCs from mouse ESCs and further differentiated the EpiLCs into PGCLCs, which were then aggregated with E12.5 gonadal somatic cells. These aggregates were cultured for approximately 5 weeks in three steps: in vitro differentiation, in vitro growth, and in vitro maturation [79,80]. The aggregates of PGCLCs and gonadal somatic cells formed a follicular structure that successfully simulated the in vivo ovarian environment and performed the key events of oogenesis in vitro, including the formation of cumulus-oocyte complexes and development of MII oocytes [79,80]. These in-vitro-generated oocytes could be fertilized in vitro with wild-type sperm and developed into 2-cell embryos, giving rise to healthy fertile pups after transfer to pseudopregnant surrogate mice. However, the successful full-term development rate was as low as 3.5%. This in-vitro-oocyte generation method was also valid for iPSC lines reprogrammed from mouse embryonic fibroblasts [79]. Overall, this platform for in vitro gametogenesis by aggregating PSC-derived PGCLCs with gonadal somatic cells faithfully reconstitutes the in vivo-like gonadal environment in both males and females [68,72,79,80]. However, this approach still shows meiosis and epigenetic imperfections compared with their in vivo counterparts and requires embryonic somatic cells [68,79], which cannot be used in the clinic. Nevertheless, this differentiation method provides a useful in vitro system for studying the interaction between gametes and gonadal somatic cells [81,82].

Recently, oocyte-like cells were directly induced without specification into PGCLCs [83]. Hamazaki et al. generated follicle structures from mouse ESCs and iPSCs aggregated with E12.5 female gonadal somatic cells through forced expression of eight transcription factors important for primordial-to-primary-follicle transition (PPT), such as *Nobox*, *Figla*, *Tbpl2*, *Sohlh1*, *Stat3*, *Dynll1*, *Sub1*, and *Lhx8*. Oocyte-like cells of the follicle structures could develop into MII oocytes, which were competent for fertilization with sperm and early embryonic development until the 8-cell stage. Overexpression of these eight transcription factors was not sufficient for the induction of oocyte-like cells from somatic cells (MEFs), suggesting that a pluripotent state is required for the induction of germ cells by overexpression of the eight transcription factors. However, it is still unknown whether different transcription factor combinations may induce germ cell specification in somatic cells.

Furthermore, follicle-like cells (FLCs) were first derived from human PSCs by forced expression of DAZL and BOULE, which are involved in in vivo germ cell specification as discussed above [54,84]. Germ cell commitment from hESCs was first induced by BMP4 and BMP8a treatment followed by meiosis induction by lentiviral overexpression of DAZL and BOULE with the subsequent addition of GDF9 and BMP15. Follicle-like structures with oocyte-like cells surrounded by multilayered cell aggregates appeared on day 9 after differentiation. These oocyte-like cells expressed ZP2, NOBOX, AMH, and VASA and were found to have primordial oocyte identity, as confirmed by transcriptome analysis. In addition, transplantation of FLCs into kidney capsules resulted in the formation of primordial follicle-like structures, indicating that hESC-derived FLCs are functional ovarian follicles. This in vitro FLC derivation system could be used to elucidate the early mechanism of human germline development and folliculogenesis, the details of which are still elusive.

## 4. Germ Cell Derivation via Synthetic Embryo Formation

In addition to direct differentiation from PSCs, synthetic embryo formation is an emerging technique for the derivation of germ cells in vitro. Aggregation of two or three types of blastocyst-derived stem cells, including ESCs, trophoblast stem cells (TSCs), and extra-embryonic endoderm (XEN) cells, can form early embryo-like structures or synthetic embryos, mimicking natural embryos [85,86,87,88]. In 2017, Zernicka-Goetz and colleagues first generated synthetic embryos, ETS-embryos, by aggregating ESCs and TSCs, which were then cultured in a 3D-extracellular matrix (ECM) scaffold [85]. ETS-embryos successfully formed a proamniotic cavity and underwent key spatiotemporal morphogenesis of peri- and post-implantation stages, as seen at E6.5 natural embryos, leading to the specification of PGCs expressing *Stella*, *Prdm14*, *Tfap2c*, *Nanos3*, and *Ddx4*. PGC specification in ETS-embryos was found to be induced by BMP-SMAD signaling, similar to natural embryogenesis.

Moreover, ETX embryos formed by the incorporation of ESCs, TSCs, and XEN cells developed to form gastruloid-like natural E7.0 mid-gastrular embryos [86]. ETX gastruloids showed more efficient mesodermal specification than ETS-embryos and asymmetric patterning, that is, anterior and posterior axis followed by PGC specification as shown by *Stella*- and *Prdm14*-positive populations at the posterior portion of the boundary of ESC and TSC compartments [86]. Similarly, ETX embryos generated under a nonadherent-suspension-shaking culture system also showed asymmetric patterning of PGC specification [87]. However, these synthetic embryos showed limited developmental potential only in the early post-implantation stage. Therefore, it is not yet known whether subsequent germline differentiation beyond early PGC specification through synthetic embryo formation is possible. However, these studies demonstrated that differentiation via synthetic embryo formation can be another way to generate PGCs from PSCs in vitro.

Recently, Liu et al. reported the successful generation of a human blastocyst-like structure (iBlastoid) via reprogramming of fibroblasts [89]. During pluripotential reprogramming, unwanted cell types, such as trophoblasts and primitive endoderm and epiblast cells, emerged in the same dish by day 21 post-induction. After 3D culture of these heterogeneous cell populations in the AggreWell system for 6 days, aggregates developed into E5–7 human blastocyst-like structures, that is, iBlastoids. Under attachment culture of iBlastoids to test peri-implantation development, iBlastoids did not show any morphological signs of implantation or gastrulation. Although human PGCs have not been derived through synthetic embryo (iBlastoid) formation, we cannot rule out the possibility of this method as an alternative method to generate PGCs in vitro by reprogramming somatic cells.

## 5. In Vitro Gametogenesis for Clinical Applications

One-fifth to one-sixth of couples suffer from infertility, which clinically means an inability to conceive even after one year or longer without contraception [90]. The causes of infertility are divided into two parts: primary and secondary. Primary sterility involves structural problems of the genital tract, such as primary ovarian insufficiency (POI), polycystic ovary syndrome (PCOS), endometriosis, and abnormal semen, whereas secondary sterility involves genetic, metabolic, and lifestyle problems [90,91]. In vitro germ cell differentiation using iPSCs derived from infertile patients may provide an in vitro system for studying the pathology and etiology of infertility at the cellular level [92].

Non-obstructive azoospermia (NOA) is a form of male infertility [93]. The main cause of NOA is genetic abnormalities, including Y chromosome microdeletions or karyotype abnormalities. Zhao et al. developed a protocol for the efficient derivation of spermatogonium-like cells from hiPSCs and applied this method to hiPSCs derived from NOA patients [94]. They differentiated hiPSCs via EB formation in feeder- and serum-free conditions, which efficiently induced PLZF^+^ spermatogonium-like cells. However, they found that hiPSCs from patients with the Sertoli cell-only syndrome produced PLZF^+^ cells less efficiently. Moreover, hiPSCs from patients with mild symptoms (due to AZFc microdeletion) showed a relatively normal germ cell differentiation efficiency [94], suggesting that evaluating the in vitro generation of spermatogonium-like cells can be used in in vitro clinical diagnosis of male infertility, such as NOA. In another set of experiments using NOA hiPSCs, in vitro hPGCLCs were induced via iMeLC formation [95]. hPGCLCs derived from NOA hiPSCs showed a significantly lower proportion of EpCAM/INTEGRINα6 (hPGCLC markers) double-positive cells than those derived from normal hiPSCs. Moreover, the expression levels of early PGC markers (*BLIMP1*, *TFAP2C*, *SOX17*, and *NANOS3*) were reduced in NOA hiPSC-derived hPGCLCs. They also found that differentiating EBs from NOA hiPSCs showed a higher percentage of apoptotic cells than normal hiPSCs. These results demonstrate that NOA hiPSCs poorly differentiate into hPGCLCs, which may be due to apoptosis during germline specification.

Botman et al. tested the germ cell differentiation potential of Klinefelter syndrome (KS) hiPSCs derived from 47 XXY-fibroblasts [96]. KS is the main genetic cause of NOA (11% of patients with azoospermia) [97]. To differentiate KS-hiPSCs into the germ line lineage, KS-hiPSCs were cultured in a medium with a combination of germline-inducing factors, including BMP4, the glial-derived neurotrophic factor (GDNF), retinoic acid, and stem cell factor (SCF), for 6 weeks. Compared with wild-type hiPSCs after 6 weeks of differentiation, KS-hiPSCs produced fewer germ cells expressing germ cell markers, BOLL and MAGEA4. They found that the reduced differentiation efficiency of KS-hiPSCs into germ cells was due to the increase in apoptosis upon germ cell differentiation, as revealed by the fact that a greater proportion of BOLL^+^ and MAGEA4^+^ cells were found to be positive for apoptosis markers, lactate dehydrogenase, and caspase-3 [96].

In 2015, Lizhi et al. explored the differentiation potential of hiPSCs derived from infertile patients with primary ovarian insufficiency (POI) with terminal Xq deletions [98]. POI-hiPSCs displayed the normal characteristics of pluripotency, such as morphology, expression of pluripotency-related genes, and differentiation potential, similar to hESCs. To monitor germ cell differentiation, POI-hiPSCs were transfected with the *VASA*-*GFP* reporter. PGC differentiation was induced by treatment with BMP4 or Wnt3a. After day 8 of the differentiation, GFP-positive cells appeared and reached a peak (approximately 6–8%) on day 12. VASA-GFP^+^ cells expressed early germ cell markers, such as *BLIMP1*, *DPPA3*, *STELLA*, and *DAZL*, whereas the meiotic marker *SCP3* was not detected, indicating that POI-hiPSCs could differentiate into premeiotic PGCs. However, these VASA-GFP^+^ cells disappeared after day 16 of the differentiation. They also found that five genes localized in the deleted Xq region in POI-hiPSCs were significantly downregulated in VASA-GFP^+^ cells derived from POI-hiPSCs [98,99]. Considering that these five genes in the deleted Xq region are associated with germ cell development, the five genes in the deleted Xq region are responsible for the etiology of POI.

## 6. Safety and Ethical Concerns

While using differentiated gametes to counter infertility as a feasible solution, safety issues must be considered before applying such technology for medical needs. These issues include genomic and epigenomic instability accumulated in PSC-derived gametes, which may pose various health concerns in patients and subsequent generations. Genomic integrity issues are no strangers when it comes to PSC culture. Prolonged cell culture or even in vivo cell growth evokes methylation profiles and epigenetic alterations [100]. While hESCs can repair various types of DNA damages in a much more efficient manner than their differentiated counterparts [101], hiPSCs under a long-term culture show deteriorated DNA repair capability, including regressed recognition of genomic imparity and coping strategies [102]. Moreover, the MEK1/2 inhibitor, which is widely used for the maintenance of pluripotency in hPSCs, is known to induce chromosomal abnormalities and erode epigenetic imprints [103]. These genetic abnormalities may cause unknown health concerns, including an increased risk of developing cancer. Passing genetic defects onto gametes is even more critical, potentially affecting every cell in the next generation with innate DNA lesions. Although the oocyte is known to correct slight DNA damage in gametes, the degree of damage compensated by the oocyte repair mechanism remains unknown [104]. The formation of gametes from defective PSCs may be the onset of impacting hereditary mutations in subsequent generations. Furthermore, the in vivo fertilization process includes natural culling of defective gametes via competition. In contrast, in vitro artificial fertilization techniques require manual selection of gametes and several steps of natural gamete selection are inevitably skipped, leaving PSC-derived germ cells exposed to genetically induced diseases [104].

Epigenetic events play a crucial role in germ cell development [105]. Sex-specific imprinting patterns are engrafted during gametogenesis after being erased via PGC migration. However, embryos generated by ART and the gametes from pluripotent stem cells show an impact on epigenetic alterations and imprinting disorders such as Beckwith–Wiedemann syndrome (BWS) [106,107]. BWS is involved in mutations and altered expression in the imprinted 11p15.5 chromosome region, especially the genes closely associated with the cell cycle and growth [108]. Approximately 75% of BWS cases are sporadic and caused by epimutation of 11p15.5 IC1 (Imprinting Center 1) and IC2. IC1 and IC2 are important insulators in a differentially methylated region (DMR) regulating the monoallelic expression of H19 and IGF2, KCNQ1OT1, and CDKN1C each [108,109,110]. Mutations in these regions cause biallelic expression, leading to fetal overgrowth, macroglossia, and anterior abdominal wall defects [108,111]. In 2003, DeBaun et al. confirmed that six of the seven children with BWS were born after IVF. Molecular studies of six children indicated that five of them had abnormal imprinting at the antisense transcript gene LIT1 and the other showed hypermethylation at H19 DMR [106]. PSC-derived gametes are also likely to create such issues [112]. Therefore, it is crucial to test the quality of PSC-derived gametes.

Ethical and social concerns should also be considered in PSC-derived germ cell applications. Derivation of germ cells from PSCs opens windows for the clinical application of germ cells to innately infertile individuals. However, inadequately utilized germ cell derivation from hiPSCs may cause social problems [113]. For example, using a single hiPSC line to supply multiple gametes could give birth to uncontrolled genetically related siblings—the same ethical concern set in by ill-advised sperm donation. Moreover, large quantities of gamete production may urge the selection of embryos for better, desirous traits giving birth to designer babies. Posthumous conception is also an issue, as hiPSCs can be sustained long after the donor has passed away. Germ cells derived from dead cells may cause new social discomfort by mixing traditional family concepts. Derivation of gender-swapped gametes from a donor cell also poses a controversy, as no social consensus has been reached on this topic. Most of the issues stated above have been regarded since the last decade as an upcoming ethical issue in ART. However, former controlled technologies, such as IVF, are now deemed safe and widely applied [113]. Likewise, human germ cell derivation from PSCs must be thoroughly evaluated and regulated to fit social guidelines.

## 7. Conclusions

**Figure 2:**
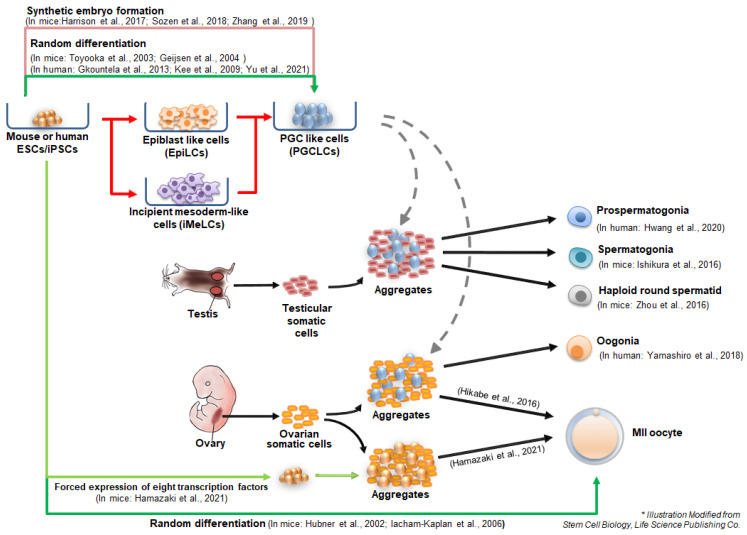
Approaches for the derivation of male and female germ cells from PSCs in vitro. PGCLCs can be derived via random differentiation, synthetic embryo formation, EpiLCs, or iMeLCs. A two-step differentiation method is the standard protocol to generate (pro)spermatogonia or mature germ cells (spermatids and MII oocytes). Modified from Stem Cell Biology, Life Science Publishing Co. [27].

Various approaches have been developed to improve the in vitro generation of male and female gametes derived from PSCs (Figure 2). Differentiating cells must undergo meiosis to generate mature germ cells, which require a special gonadal environment in vivo. Currently, the two-step differentiation method (PGC specification and meiosis induction) has become the gold standard for the generation of in vitro post-meiotic germ cells. To provide an in vivo gonad-like niche, premeiotic germ cells (PGCLCs) were aggregated with testicular or ovarian somatic cells, from which haploid spermatids and MII oocytes could be successfully derived [79,114]. As a pioneering approach, direct induction of germ cells by forced expression of transcription factors has also been attempted. As direct conversion from a somatic cell type to another type of somatic cell is possible in certain cases [115], direct conversion of somatic cells into germ cells could also be applied. Medrano et al., in 2016, transduced various genes (PRDM1, PRDM14, LIN28A, DAZL, VASA, and SYCP3: i6F conditions), resulting in the cell clumps expressing upregulated human PGC markers, claiming direct conversion of human fibroblasts to germ-like cells [116]. However, the strategy of Hamazaki and colleagues to generate MII oocytes using the transduction of eight transcription factors has not been successful in differentiated somatic cells [83].

Recently, several advanced germ cell specification models have been widely applied to understand the mechanism of germ cell induction from the pluripotency state. Using a T-induction system, Aramaki et al. confirmed the correlation between T and pluripotency core genes in early germ cell fate progression in PSCs [117]. Moreover, intermediate PSC states competent for both chimeras and PGCs were developed from several species, including humans, providing another differentiation source for direct PGC specification without transient progenitors [118]. Such progress aids in a detailed reconstitution method, further increasing the efficiency of germline derivation from PSCs. Moreover, in vitro gametogenesis platforms could be utilized to determine and compensate for the causes of infertility using germ cells from patient-origin hiPSCs (NOA and POI), thereby leading to an increase in successful fertility rates [94,95,96,98]. In vitro gametogenesis can be used to understand the biological mechanism of gamete development and to treat infertile couples. Derivation of functional spermatozoa or MII oocytes from human iPSCs is currently not possible. However, if mature spermatozoa and oocytes can be derived from patient (NOA or POI) cells, these in vitro-derived gametes will change the strategy of current assisted reproductive technology (ART) and in vitro sperm and oocytes can be directly used for IVF and embryo transfer to give birth to babies without ethical issues of germ cell donation. In addition, patient cell-derived oocytes can be used to rejuvenate or induce healthy oocytes and boost fertility by transferring ooplasm to old or low-quality oocytes [119]. For example, cytoplasts from hPSCs could be another therapeutic source for patients with mitochondrial DNA mutations, which is one of the causes of developmental arrest in embryos [120]. This approach is currently feasible because early germ cells, such as oogonia, can be used as ooplasm or mitochondrial donors [121].

## Data Availability

The full data set can be provided by the authors upon request.
